# Effect of Replacing in-Feed Antibiotic Growth Promoters with a Combination of Egg Immunoglobulins and Phytomolecules on the Performance, Serum Immunity, and Intestinal Health of Weaned Pigs Challenged with *Escherichia coli* K88

**DOI:** 10.3390/ani11051292

**Published:** 2021-04-30

**Authors:** Yunsheng Han, Tengfei Zhan, Chaohua Tang, Qingyu Zhao, Dieudonné M. Dansou, Yanan Yu, Fellipe F. Barbosa, Junmin Zhang

**Affiliations:** 1 State Key Laboratory of Animal Nutrition, Institute of Animal Sciences of Chinese Academy of Agricultural Sciences, No. 2 Yuan Ming Yuan West Road, Beijing 100193, China; hanyunshengcaas@163.com (Y.H.); zhantf2019@sina.com (T.Z.); tangchaohua@caas.cn (C.T.); zhaoqingyu@sina.com (Q.Z.); donnedansou@outlook.com (D.M.D.); yuyanan@caas.cn (Y.Y.); 2 Scientific Observing and Experiment Station of Animal Genetic Resources and Nutrition in North China of Ministry of Agriculture and Rural Affairs, Institute of Animal Science, Chinese Academy of Agricultural Sciences, Beijing 100193, China; 3 EW Nutrition GmbH, Visbek, Court of Registration: AG Oldenburg HRB 200104, Hogenbögen 1, 49429 Visbek, Germany

**Keywords:** feed conversion ratio, post-weaning diarrhea, serum immunoglobulins, host inflammation, intestinal morphology, fecal coliforms

## Abstract

**Simple Summary:**

Post-weaning diarrhea (PWD) in pigs caused by *Escherichia coli* (*E. coli*) is a global problem which results in substantial economic losses, due to decreased performance and a high incidence of mortality and morbidity. Due to the banning of antibiotic growth promoters (AGPs) by many countries, it would be valuable to find environmentally friendly and non-antibiotic alternatives to AGPs and to evaluate their effectiveness. Both immunoglobulins and phytomolecules are separately reported as benefiting animal growth, but the efficiency of combinations of immunoglobulins and phytomolecules as AGP alternatives is largely unknown. In this study, the results showed that a mixture of immunoglobulin and phytomolecule administration had positive effects on feed efficiency, diarrhea reduction, intestinal morphology, and coliform control. Combinations of immunoglobulins and phytomolecules can be used as a potential alternative to AGPs in weanling piglets.

**Abstract:**

The study was conducted to investigate the effects of replacing antibiotic growth promoters (AGPs) with an egg immunoglobulin (IgY) combined with phytomolecules (PM) on the growth rate, serum immunity, and intestinal health of weaned pigs challenged with *Escherichia coli* K88 (*E. coli* K88). A total of 192 piglets were weaned at 28 days old with an average weight of 7.29 (± 0.04) kg. They were randomly divided into four treatments containing eight replicates with six piglets per replicate. The treatment groups were NC and PC fed a basal diet, AGP fed a basal diet supplemented with 75 mg/kg chlortetracycline, 50 mg/kg oxytetracycline calcium, and 40 mg/kg zinc bacitracin, IPM fed a basal diet supplemented with IgY at dose of 2.5 g/kg and 1.0 g/kg and PM at dose of 300 mg/kg and 150 mg/kg during days 1 to 17 and 18 to 42, respectively. On days 7 to 9 of the experiment, piglets in the PC, AGP, and IPM groups were orally challenged with 20 mL *E. coli* K88 (10^9^ CFU/mL), while piglets in the NC group were challenged with 20 mL medium without *E. coli* K88. The *E. coli* K88 challenge model was successful as the incidence of post-weaning diarrhea (PWD) of piglets challenged with *E. coli* K88 was significantly higher than that of those unchallenged piglets during the challenge time (days 7 to 9) and days 1 to 7 of post-challenge (*p* < 0.05). A diet with combinations of IgY and PM and AGPs significantly decreased the incidence of PWD during the challenge time and days 1 to 7 of post-challenge (*p* < 0.05) compared to the PC group and significantly improved the ratio of feed to weight gain (F:G) during days 1 to 17 of the experiment compared to the NC and PC groups (*p* < 0.05). In comparison with the PC group, piglets in the IPM group had significantly higher serum levels of IgA, IgG, and IgM (*p* < 0.05), but lower serum IL-1β on day 17 of experiement (*p* < 0.05). Besides, diet supplementation with AGP significantly decreased serum IL-1β, IL-6, and TNF-α on days 17 and 42 (*p* < 0.05) with comparison to the PC group. Piglets in the IPM group showed a significantly lower level of fecal coliforms (*p* < 0.05), but a higher villus height of jejunum and ileum and higher ratio of villus height to crypt depth of duodenum and jejunum (*p* < 0.05) than those piglets in the PC group. In summary, diet supplementation with a mixture of IgY and PM decreased the incidence of PWD and coliforms, increased feed conversion ratio, and improved intestinal histology and immune function.

## 1. Introduction

Weaning is a critical period in pig production because piglets are subjected to several changes such as a new feeding system and different housing. The stress on an immature immune system caused by such changes increases the susceptibility to pathogens. These pathogens may overgrow in the gastrointestinal tract leading to clinical diseases, such as post-weaning diarrhea (PWD), which may compromise feed intake and growth rates. In-feed antibiotic growth promoters (AGPs) have been used extensively as the solution for a less stressful transition through the weaning period, effectively reducing the incidence of PWD and improving the growth performance of livestock [1,2]. However, several reports indicated that the prophylactic use of antibiotics markedly increased the bacterial resistance to antibiotics [3,4,5,6], so AGPs have been banned in many countries [2], including China in 2020.

Egg yolk immunoglobulins (IgY) have been successfully used in weaned piglets’ nutrition for several years, and their main goal was supporting piglets by creating a more stable gut microbiota [7,8]. These immunoglobulins or antibodies are obtained from eggs of laying hens immunized with a specific bacterial antigen and have an inhibitory effect on homologous pathogenic bacteria [9,10]. In vivo and in vitro studies demonstrated that specific IgY can reduce diarrhea caused by *Salmonella*, rotavirus, and *E. coli* and enhance piglet’s immunity and growth performance [11,12,13,14].

Phytomolecules (PM) are bioactive compounds derived from plants, including carvacrol, cinnamaldehyde, and capsaicin. Many PM have antibacterial, anti-inflammatory, and antioxidant properties [15,16]. Several studies demonstrated that these biological activities benefit the intestinal health and growth performance of pigs [17,18,19]. A possible strategy to increase the efficiency of alternatives is to adopt a combinatorial strategy, taking advantage of the synergy and complementation of the available products. Both IgY and PM have been reported as benefiting domesticated animal growth and health [17,19,20], but the efficiency of the mixture of IgY and PM as an AGP alternative is unrevealed.

In this study, we hypothesized that combinations of IgY and PM might improve intestinal function, and subsequently contributing to weanling piglet’s growth and health. *Escherichia coli* (*E. coli*) has been described as one of the most important pathogens for piglets suffering PWD [21,22,23,24], causing severe inflammatory processes and disrupting gut morphology, which can increase morbidity and mortality of piglets [24,25,26,27]. Moreover, previous studies have used piglets challenged with *E. coli* K88 as a weaning stress model [27,28]. Thus, the present study used an *E. coli* K88 challenging model in piglets to evaluate the effects of combination of IgY and PM as alternatives to AGP on the growth performance, serum immunity, and intestinal health of weaned piglets. It aimed to provide a theoretical basis for the application of mixtures of egg immunoglobulins and phytomolecules replacing antibiotics in swine production.

## 2. Materials and Methods

All experimental procedures followed the recommendations of “Guidelines on Welfare and Ethical Review for Laboratory Animals” (GB/T 35892-2018) [29] approved by the Experimental Animal Welfare and Ethics Committee of Institute of Animal Sciences of Chinese Academy of Agricultural Sciences (IAS 2019-77).

### 2.1. Bacteria, Alternative and Antibiotic Growth Promoters

The strain of *E. coli* K88 (O8: K87, K88ac) was obtained from China Veterinary Culture Collection Center (Beijing, China). The *E. coli* K88 strain was grown in LB medium (Beijing Land Bridge Technology Co., Beijing, China) aerobically at 37 °C overnight. The techniques for culturing this strain were based on the descriptions of Marquardt et al. [26]. Two commercial additives Globigen^®^ Jump Start and Activo^®^ (IgY and PM), were provided by EW Nutrition GmbH (Visbek, Germany) as alternatives. According to the manufacturer, IgY is an egg powder containing natural immunoglobulins to support the intestinal health of young animals, while PM is a microencapsulated feed additive, with a mixture of phytomolecules that promote intestinal health through antibacterial, digestive and antioxidant activities. The AGPs premix consisting of 75 g chlortetracycline, 50 g oxytetracycline calcium and 40 g zinc bacitracin per kilogram was provided by Inner Mongolia Xingdaye Agriculture and Animal Husbandry Company Limited (Wulanchabu, Inner Mongolia, China).

### 2.2. Experimental Set-Up, Animals, Diets and Challenge

A total of 192 “Duroc × Landrace × Great White” weaned pigs, aged 28-day-old with an equal sex ratio, comparable general health, and uniform mean body weight of 7.29 (±0.04) kg were randomly assigned to four treatment groups. Each treatment group had eight replicates (pens) with six piglets per pen. The piglets were group-housed in the same pigsty with a double curtain side and a hard plastic fully slotted floor. Room temperature was controlled by an electric air curtain and kept at 30 °C during days 1 to 7, then at 28 °C during days 8 to 17, and finally at 26 °C during days 18 to 42.

Two phase basal diets were formulated to meet the National Research Council (2012) [30] nutrient requirements (Table 1). The negative control (NC) and the positive control (PC) groups were given the basal diet and the AGPs supplemented (AGP) group was given the basal diet supplemented with 75 mg/kg chlortetracycline, 50 mg/kg oxytetracycline calcium, and 40 mg/kg zinc bacitracin. The IgY combined with PM-supplementation (IPM) group was given the basal diet supplemented with IgY at a dose of 2.5 g/kg and 1.0 g/kg and PM at a dose of 300 mg/kg and 150 mg/kg during days 1 to 17 and 18 to 42, respectively. On days seven, eight and nine of this experiment, piglets in the PC, AGP, and IPM groups were orally challenged with 20 mL *E. coli* K88 at a concentration of 10^9^ CFU/mL, while piglets in the NC group were orally challenged with 20 mL medium without *E. coli* K88. This trial lasted for 42 days. Feed and water were given ad libitum.

### 2.3. Sample Collection and Parameter Analysis

#### 2.3.1. Growth Performance

On days 1, 17, and 42 of the experiment, body weight (BW) and feed consumption were recorded on pen basis to calculate average daily gain (ADG), average daily feed intake (ADFI), and feed intake to weight gain ratio (F:G).

#### 2.3.2. Post-Weaning Diarrhea Incidence

Throughout the experiment, fecal scores were visually assessed for each piglet every morning by the same observer who was blinded to the treatment groups. To perform this, a four-grade scoring system (1 = normally shaped feces, 2 = shapeless feces, 3 = soft feces, 4 = thin, liquid feces) was used and piglets with scores greater than 3 were assigned as having diarrhea [31]. Based on the fecal scores for each piglet, daily numbers of piglets with diarrhea were recorded per replicate. Then, the incidence of diarrhea from day 1 to 6 (period before challenge), day 7 to 9 (challenge time), day 10 to 16 (day 1 to 7 post-challenge), day 17 to 26 (day 8 to 17 post-challenge), and day 27 to 42 of the experiment (day 18 to 33 post-challenge) were respectively calculated using the following formula: the incidence of diarrhea = (diarrhea piglets × diarrhea days)/(total piglets × experimental days) × 100%. The data of average incidence of diarrhea during each experimental period was used for further statistical analysis.

#### 2.3.3. Serum Immune Parameters

On days 17 and 42, one male piglet representing the average weight from each replicate was selected for a 10 mL blood sample to be collected from the precaval vein into coagulative vacuum tubes. The samples were placed on ice for two hours and then centrifuged at 3000× *g* for 10 min at 4 °C and serum was stored at −20 °C. Serum was used for detecting immunoglobulins IgA, IgM, and IgG, interleukins IL-1β and IL-6 and tumor necrosis factor (TNF-α). These parameters were measured using an ELISA kit (Shanghai Enzyme-linked Biotechnology, Co., Ltd., Shanghai, China) according to the manufacturers’ protocols.

#### 2.3.4. Intestinal Morphology

On day 17, after fasting overnight, one male piglet representing the average weight from each replicate was chosen and anesthetized by intramuscular injection of anesthetics including zolazepam and tiletamine at a dosage of 0.1 mg/kg BW. Under full anesthesia, the piglet was humanely slaughtered. The intestine was divided into three parts including duodenum, jejunum, and ileum, and a middle section of these three segments with two cm was respectively collected, slighted washed by saline, and fixed in a 4% phosphate buffered formalin solution. The intestinal specimens were embedded in paraffin and four µm of tissue sections were prepared and stained with hematoxylin, eosin, and periodic acid-Schiff. Three randomly oriented villi and their adjoining crypts in each sample were selected and blindly examined using an image analysis system (Version 1, Leica Imaging Systems Ltd., Cambridge, UK) [32]. Villus height (VH) and their adjoining crypt depth (CD) were measured and the ratio of VH to CD (V/C) was calculated from these measurements.

#### 2.3.5. Fecal Coliforms Bacteria

After euthanasia of piglets, fresh and clean feces samples were collected through rectum and coliforms were counted after culture using MacConkey medium according to the method described by a previous study [33]. Briefly, nine mL sterilized saline liquor was added to 1.0 g of each fecal sample for dilution, mixed evenly and then diluted to 10^−2^, 10^−4^ and 10^−6^ g/mL, with 100 µL of the homogenates plated onto MacConkey medium (Beijing Land Bridge Technology Co., Beijing, China) and cultured at 37 °C for 24 h under aerobic conditions. All data were performed in triplicate.

### 2.4. Statistical Analysis

SPSS version 22.0 (IBM Corp., Armonk, NY, USA) was used to perform the analyzes. Data were analyzed with one-way ANOVA and Duncan’s multiple-range test was used to analyze variations between treatments for growth performance, the incidence of PWD, serum immune indexes, fecal coliforms, and intestinal morphology. Results of fecal coliforms were presented as log_10_ CFU/g feces for further statistical analysis. *p* < 0.05 indicated significant differences.

## 3. Results

### 3.1. Growth Performance

Dietary supplementation with combination of IgY and PM increased feed conversion ratio by significantly improving F:G ratio during days 1 to 17 compared to the NC and PC groups (*p* < 0.05) as seen in Table 2. The F:G ratio in the AGP group was significantly lower than that in the PC group during days 1 to 17 and 1 to 42 (*p* < 0.05). No difference in F:G ratio was observed between the IPM and AGP groups (*p* > 0.05). There was no significant difference in ADG and ADFI among the NC, PC, AGP, and IPM groups (*p* > 0.05).

### 3.2. Post-Weaning Diarrhea Incidence

In the present study, all treatment groups were presented in each replicate under blind evaluation. Compared with piglets in the NC group, *E. coli* K88 challenge significantly increased the incidence of PWD on piglets from the PC and IPM groups during days 7 to 9 of the experiment (challenging time) (*p* < 0.05), and on piglets from the PC, AGP, and IPM groups during days 1 to 7 post-challenge (*p* < 0.05) (Table 3). The incidence of PWD on piglets from the IPM and AGP groups during challenging time were significantly lower (*p* < 0.05), and on piglets from the IPM group during days 1 to 7 post-challenge was significantly lower (*p* < 0.05), than that in the PC group. Despite the decrease trend of the incidence of PWD on piglets in the AGP and NC groups during days 8 to 17 post-challenge compared with the PC group (*p* = 0.061), no significant difference was found among the NC, PC, AGP, and IPM groups during days 8 to 17 and 18 to 33 post-challenge (*p* > 0.05). These results indicated that the *E. coli* K88 challenge model was successful and dietary combinations of IgY and PM effectively decreased incidence of PWD caused by *E. coli* K88.

### 3.3. Serum Immune Parameters

The effects of dietary treatments on serum immune indices of weaned pigs challenged with *E. coli* K88 are presented in Table 4. On day 17 of the experiment, serum levels of IL-1β, IL-6, and TNF-α in the PC group were significantly higher, but serum levels of IgA, IgG, and IgM in the PC group were significantly lower, than that in the NC groups (*p* < 0.05). Serum level of IgG on day 42 was significantly lower than that in the NC and AGP groups (*p* < 0.05). Compared with the PC group, serum levels of IgA, IgG, and IgM on day 17 and 42 in the IPM group were significantly increased (*p* < 0.05), but serum level of IL-1β on day 17 was significantly decreased (*p* < 0.05). There was no significant difference in serum levels of IL-1β, TNF-α, and IgM on day 17 and serum level of TNF-α, IgG, and IgM on day 42 among the NC, AGP and IPM groups (*p* > 0.05). The serum IL-6 level on days 17 and 42 and serum IL-1β level on day 42 in the IPM group were significantly higher than those in the AGP group (*p* < 0.05), but no significant differences were found between the IPM and NC groups (*p* > 0.05). Serum levels of IgA and IgG on day 17 and serum IgA level on day 42 in the IPM group were significantly higher than those in the AGP group (*p* < 0.05).

### 3.4. Intestinal Morphology

The challenge with *E. coli* K88 negatively affected intestinal morphology by significantly decreasing the V/C of duodenum and jejunum (*p* < 0.05) and decreasing the VH of three intestinal segments (*p* > 0.05) compared with the NC group (Table 5). In comparison with the PC group, dietary with IgY and PM significantly increased VH and V/C of jejunum and VH of ileum (*p* < 0.05). The AGP group had higher VH and V/C of duodenum and had higher VH of ileum (*p* < 0.05). For the jejunum segment, the VH in the IPM group was higher than that in the AGP group (*p* < 0.05), and the V/C in the IPM group was significantly higher than that in the NC and AGP groups (*p* < 0.05).

### 3.5. Fecal Coliform Bacteria

**Figure 1:**
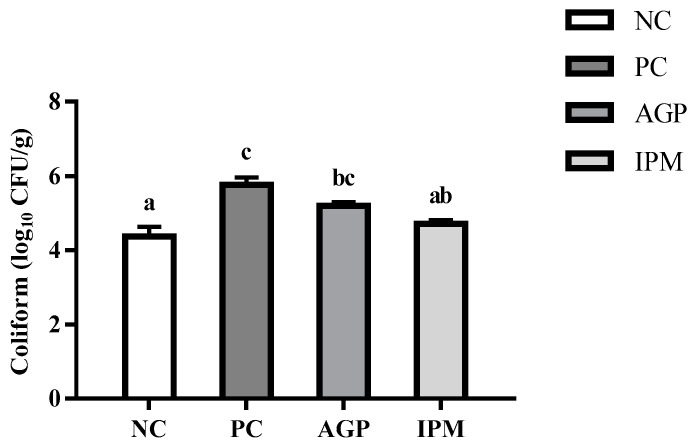
Effect of dietary treatments on fecal coliforms of *E. coli* K88-challenged weaned pigs. ^a–c^ means within a same pattern with no common superscript indicate significant difference between groups (*p* < 0.05). Results were presented as log_10_ CFU/g feces and as the mean values and the standard error of the mean, (*n* = 8). Dietary treatments were as follows: NC, negative control group, basal diet; PC, positive control group, basal diet, and challenged with *E. coli* K88; AGP, antibiotic growth promoter group, basal diet supplemented with 75 mg/kg chlortetracycline, 50 mg/kg oxytetracycline calcium, and 40 mg/kg zinc bacitracin, and challenged with *E. coli* K88; IPM, IgY and PM group, basal diet supplemented with IgY at dose of 2.5 g/kg and 1 g/kg and PM at dose of 300 mg/kg and 150 mg/kg during days 1 to 17 and 18 to 42, respectively, and challenge with *E. coli* K88.

The PC and AGP groups had a greater fecal coliform bacteria numbers than the NC group (*p* < 0.05, Figure 1), which further demonstrated the success of this *E. coli* K88 challenge model. Compared with the PC group, the coliform bacteria in the IMP group was significantly decreased (*p* < 0.05), but no significant difference was found between the IMP and AGP groups (*p* > 0.05).

## 4. Discussion

Weaning stress is generally associated with a decrease in feed intake and feed conversion ratio as well as nutritional, social, and immunological disorders which negatively affect the growth performance of piglets. The present study employed an *E. coli* K88-induced weaning-like stress model to evaluate the effects of different combinations of IgY and PM as AGPs alternatives on growth performance and health of weaned pigs [27,28]. In recent years, IgY and PM have been separately applied in livestock production, while the effects of combined IgY and PM on weaning piglets challenged with *E. coli* k88 were not reported. Marquardt et al. observed dietary administration with IgY increased the weight gain of early weaned pigs challenged with *E. coli* K88 [26]. Owusu-Asiedu et al. reported that the combination of IgY and a highly digestible pea protein improved weight gain and feed intake of weanling piglets [11]. Essential oils, one sort of PM, were extracted from eucalyptus, *Petroselinum crispum* and thyme, which demonstrated that diet supplemented with these at levels of 150 mg/kg or 200 mg/kg had a similar growth promoting effect to AGPs [34]. Carvacrol, cinnamaldehyde and capsicum oleoresin used in piglet diets alone or as combinations could effectively promote animal growth performance [35,36,37,38,39]. These studies agreed with the present results that diets with combinations of IgY and PM significantly increased the feed conversion ratio of weaned piglets challenged with *E. coli* K88. This study hypothesized that the increase in feed conversion ratio might be related to intestinal absorption, function, and gut health improvement.

Weaning stress generally causes a considerable incidence of PWD in piglets, which results in an increase in morbidity and mortality and serious economic losses [24,25,40]. In the present study, the incidence of PWD was monitored to evaluate the effectiveness of combined IgY and PM in preventing *E. coli* K88 induced-diarrhea. The present study indicated that dietary supplementation with combined IgY and PM significantly diminished the risk of PWD during days 7 to 9 of challenging time and days 1 to 7 post-challenging of experiment. In accordance with the current study, previous studies reported that the use of IgY could reduce the incidence of diarrhea of weaned piglets challenged with toxicogenic *E. coli* [11,26,41]. The clinical symptoms observed for piglets suggested that IgY-treated piglets recovering from *E. coli* K88 induced-diarrhea was faster than piglets untreated with IgY [42]. Moreover, dietary supplementation with a mixture of plant essential oils, eucalyptus essential oil, *Petroselinum crispum* essential oil, and thyme essential oil had a similar anti-diarrheal effect to AGPs [34]. These results indicated that supplementation with a combination of IgY and PM could reduce the incidence of PWD when piglets are challenged with *E. coli* K88.

Worldwide, *E. coli* is considered as one of the most critical pathogens for pigs in the weaning period due to its ability to adhere to the epithelium of the intestine and secrete enterotoxins [21,23]. Coliform bacteria were counted in the present study to explore the potential mechanism of combined IgY and PM decreasing the incidence of PWD of weaned piglets challenged with *E. coli* K88. The combination of cinnamaldehyde essential oils and thymol essential oils was previously reported as significantly reducing fecal *E. coli* [36]. Capsaicin, cinnamaldehyde, carvacrol, and 1–8-eucalyptininclusive in some plant tissues can effectively inhibit the activity of pathogenic bacteria such as *E. coli*, *Salmonella* and *Staphylococcus aureus* [43,44,45,46,47]. The mechanism underlying the control of pathogenic bacteria by PM was associated with its inhibition of the formation of biofilms and changes in the permeability of cell membranes with the loss of intracellular components and the disruption of their amino acid, lipid, and energy metabolism [18,45,48,49]. These findings support the current study, where diet supplemented with combined IgY and PM significantly decreased coliform bacteria amount when compared with the PC group. However, the antibacterial effect of IgY may be related to its ability to prevent the adhesion of pathogenic bacteria in the intestine [9,12]. IgY was found to significantly block *E. coli* K88 to bind to porcine small intestinal mucus [42]. This blocking was effective because IgY can bind to the components exposed on the surface of *E. coli* K88 resulting in structural alterations, which might impede its adhesion to intestine [50,51]. In addition, a significant decrease of heat-stable enterotoxin expression in colonic chyme was also observed in IgY-treated group at 72 h after *E. coli* infection compared with untreated group [42]. The control of *E. coli* K88-induced incidence of PWD by IgY was mainly through the restriction of *E. coli* K88 adhesion to intestinal mucus and the reduction of its enterotoxin expression. This was different from the dominant mechanism of PM decreasing the incidence of PWD, which was through sterilization or bacteriostasis. Therefore, dietary supplementation with combination of IgY and PM could effectively control the incidence of PWD of weaned piglets challenged with *E coli* K88, which might have resulted from the synergistic effect of IgY and PM.

Intestinal morphology is commonly considered an important mechanical defense of barrier function, and its features mainly including villus height and crypt depth could be a pivotal indicator of intestinal health and absorptive capacity [52,53]. Any perturbation of intestinal morphology can result in severe health issues, because it is the major route of entrance for pathogenic bacteria into the body [54]. *E. coli* infections are characterized by *E. coli* adherent to villous epithelium, and generally with villous atrophy [55]. Enterotoxins produced by pathogens like *E. coli* could result in negative effects on the intestinal morphology [22,23,24,27,56]. These were consistent with the present study that *E. coli* challenge significantly decreased the VH and V/C of the small intestine. In the present study, diets combined with IgY and PM seemed to reverse this negative trend by significantly increasing the VH and V/C of jejunum and ileum. Meanwhile, the VH and V/C of jejunum in the IPM group was even higher than that in the AGP group. The improvement of the intestinal morphology might be related to the combination of IgY and PM which effectively control *E. coli* K88 population and its enterotoxins [36,42]. This was considered with the present results that combined IgY and PM significantly increased feed conversion ratio but decreased the incidence of PWD, which benefited from the improved absorption and barrier function.

Serum IgA, IgM, and IgG are important immunoglobulins in humoral immunity. In particular, IgG has been observed in high concentrations in the serum and to have a long half-life in response to external infection [57]. The decrease in serum IgG concentration usually appear during the weaning period due to weaning stress and the immaturity of piglet immune system [58]. The present results showed that *E. coli* K88 challenge significantly decreased serum levels of IgA, IgG, and IgM on day 17 and decreased IgG level on day 42, compared to the NC group. Dietary supplementation with combination of IgY and PM reversed the immunoglobulin decrease caused by *E. coli* K88. Consistent with the present study, a previous study indicated that calves fed with 45 mg/d IgY increased their serum levels of IgG, IgM and IgA [59], probably due to passive immunization [60]. Another reason might be related to the stimulation effect of IgY on lymphocytes in blood of *E. coli*-challenged piglets [61]. In addition, dietary supplementation with a mixture of essential oils maintained the serum levels of IgM and IgA of weaned pigs at levels similar to AGP [34]. These results indicated that diets combined with IgY and PM increased serum levels of immunoglobulins under *E. coli* K88 challenge.

Cytokines are also involved in maintaining immunity homeostasis. IL-1β produced by macrophages and enterocytes is usually considered as a major inflammatory mediator in mammals, which contributes to pathogen exclusion by increasing the margination of lymphocytes [62,63]. IL-6 and TNF-α are the typical pro-inflammatory cytokines and used as potential markers to evaluate pathogen infections in piglets [64]. In the current study, *E. coli* K88 challenge significantly increased pro-inflammatory cytokines IL-1β, IL-6, and TNF-α on day 17, which then normalized by day 42. This might be equated to the disruption of intestinal morphology resulting from *E. coli* K88 challenge [28]. As reported, IgY administration could effectively suppress *E. coli*-induced inflammatory cytokines *IL-6* and *TNF-α* upregulation in intestine of piglets [42]. In a mouse model of *Salmonella typhimurium* infection, IgY supplementation reduced the expression of pro-inflammatory cytokines TNF-α and IFN-γ [14]. This research also found that IgY prevented the mobilization of T cells in the epithelium and lamina propria stimulated by *Salmonella typhimurium*. These studies might indicate that IgY supplementation can alleviate host inflammation by controlling pathogens. Moreover, dietary administration with capsaicin significantly reduced serum levels of haptoglobin and TNF-α in weaned pigs challenged with *E. coli* [37]. The probable mechanism was due to PM suppression of the nuclear factor kappa-B signal pathway and then decreased pro-inflammatory cytokines [65,66]. These results are consistent with the current study showing that a mixture of IgY and PM administration could effectively decrease pro-inflammatory cytokines production induced by *E. coli* K88. Therefore, combination of IgY and PM alleviating *E coli* K88-induced inflammation may present synergistic effects in controlling *E coli* K88 and inhibiting inflammatory signal pathway, which ensures piglets’ health during the weaning period.

## 5. Conclusions

The results of this study suggest that dietary supplementation with combinations of egg immunoglobulins and phytomolecules increases feed efficiency of weaned pigs, decreases incidence of post-weaning diarrhea and improves immunity function by controlling coliforms and improving the intestinal morphology of weaned pigs. Combinations of egg immunoglobulins and phytomolecules could protect weanling piglets from *E. coli* K88 infection to almost the same extent as AGP and therefore have potential as alternatives for weaning piglets. Such a combination applied to young weaned piglets might contribute to their health improvement. However, more high-standard research is needed to provide evidence from the perspective of intestinal microbiota and its metabolites, immune homeostasis, and interaction between microbiota and immune homeostasis.

## Figures and Tables

**Table 1:** Ingredients and chemical compositions of the basal diets (as-fed basis).

Ingredients, %	Day 1–17	Day 18–42
Extruded corn	56.50	64.00
Soybean meal	3.00	12.00
Extruded soybean	11.50	10.00
Fish meal	6.00	2.00
Soybean protein concentrate	8.00	3.00
Whey powder	11.00	5.00
Glucose	1.00	1.00
Dicalcium phosphate	0.90	1.10
Limestone	0.30	0.60
Salt	0.27	0.30
L-Lysine HCl	0.36	0.37
DL-Methionine	0.17	0.15
L-Threonine	0.16	0.18
L-Tryptophan	0.03	0.04
Vitamin and mineral premix ^1^	1.00	1.00
Nutrient composition ^2^, %		
Digestible Energy, MJ.kg^−1^	14.35	14.17
Crude protein	20.00	18.02
Ether extract	6.12	5.10
Crude Fiber	2.02	2.35
Calcium	0.88	0.78
Total phosphorus	0.76	0.66
Digestible phosphorus	0.55	0.41
Na	0.30	0.22
Cl	0.40	0.30
SID lysine	1.35	1.23
SID methionine	0.39	0.36
SID threonine	0.80	0.74
SID tryptophan	0.23	0.22

^1^ Provided per kg of diet: Vitamin A, 12,000 IU; Vitamin E, 30 IU; Vitamin D3, 2,500 IU; Vitamin B1, 1.5 mg; Vitamin B6, 3 mg; Vitamin B12, 0.012 mg; Vitamin K3, 3 mg; Choline chloride, 400 mg; Niacin, 40 mg; Riboflavin, 4 mg; Pantothenic acid, 15 mg; Folic acid, 0.7 mg; Biotin, 0.1 mg; Cu, 225 mg; Zn, 105 mg; Fe, 84 mg; Mn, 22 mg; I, 0.5 mg; Se, 0.35 mg. ^2^ Nutrient contents are calculated.

**Table 2:** Effect of dietary treatments on the growth performance of weaned pigs challenged with *E. coli* K88.

Items	Dietary Treatments ^1^				SEM ^2^	*p*-Value
	NC	PC	AGP	IPM		
Body weight, kg						
Initial	7.29	7.27	7.30	7.31	0.01	0.174
Day 17	9.57	9.42	9.77	9.80	0.07	0.131
Day 42	20.41	19.77	20.46	20.56	0.17	0.372
Days 1–17						
ADFI ^3^, g	293.23	281.55	275.52	275.52	3.61	0.267
ADG ^3^, g	142.58	133.92	154.04	155.47	4.04	0.192
F:G ^3^	2.10 ^a^	2.13 ^a^	1.82 ^b^	1.78 ^b^	0.05	0.005
Days 18–42						
ADFI, g	731.25	705.60	706.83	721.64	8.65	0.697
ADG, g	416.73	396.92	413.36	411.16	5.22	0.574
F:G	1.76	1.78	1.72	1.76	0.02	0.595
Days 1–42						
ADFI, g	564.39	544.06	542.52	551.69	5.86	0.557
ADG, g	312.29	296.73	314.57	313.75	3.87	0.321
F:G	1.81 ^a,b^	1.84 ^a^	1.73 ^b^	1.76 ^a,b^	0.02	0.098

^a,b^ Different superscript letters within a row indicate significant difference between groups (*p* < 0.05). ^1^ Dietary treatments were as follows: NC, negative control group, basal diet; PC, positive control group, basal diet, and challenged with *E. coli* K88; AGP, antibiotic growth promoter group, basal diet supplemented with 75 mg/kg chlortetracycline, 50 mg/kg oxytetracycline calcium, and 40 mg/kg zinc bacitracin, and challenged with *E. coli* K88; IPM, IgY and PM group, basal diet supplemented with IgY at dose of 2.5 g/kg and 1 g/kg and PM at dose of 300 mg/kg and 150 mg/kg during days 1 to 17 and 18 to 42, respectively, and challenge with *E. coli* K88. ^2^ SEM, standard error of the mean, *n* = 8. ^3^ ADFI, average daily feed intake; ADG, average daily gain; F:G, ratio of feed to weight gain.

**Table 3:** Effect of dietary treatments on the post-weaning diarrhea incidence of weaned pigs challenged with *E. coli K88* (%).

Items ^3^	Dietary treatments ^1^				SEM ^2^	*p*-Value
	NC	PC	AGP	IPM		
Day 1–6 b.c.	5.56	5.55	3.47	5.20	0.67	0.335
Day 7–9 c.t.	16.67 ^c^	45.23 ^a^	23.61 ^b,c^	30.55 ^b^	2.65	<0.001
Day 1–7 p.c.	25.30 ^c^	60.88 ^a^	40.21 ^a,b^	38.09 ^b^	2.48	<0.001
Day 8–17 p.c.	18.05	26.00	18.81	22.50	1.37	0.061
Day 18–33 p.c.	15.62	21.00	15.41	18.96	1.49	0.247

^a–c^ Different superscript letters within a row indicate significant difference between groups (*p* < 0.05). ^1^ Dietary treatments were as follows: NC, negative control group, basal diet; PC, positive control group, basal diet, and challenged with *E. coli* K88; AGP, antibiotic growth promoter group, basal diet supplemented with 75 mg/kg chlortetracycline, 50 mg/kg oxytetracycline calcium, and 40 mg/kg zinc bacitracin, and challenged with *E. coli* K88; IPM, IgY and PM group, basal diet supplemented with IgY at dose of 2.5 g/kg and 1 g/kg and PM at dose of 300 mg/kg and 150 mg/kg during days 1 to 17 and 18 to 42, respectively, and challenge with *E. coli* K88. ^2^ SEM, standard error of the mean, *n* = 8. ^3^ Items: Day 1–6 b.c., days 1–6 before-chanllenging with *E. coli* K88; Day 7–9 c.t., days 7–9 challenging time of experiment; Days 1–7 p.c., days1–7 post-challenging with *E. coli* K88.

**Table 4:** Effect of dietary treatments on serum immune indices of weaned pigs challenged with *E. coli* K88.

Items	Dietary Treatments ^1^				SEM ^2^	*p*-Value
	NC	PC	AGP	IPM		
Day 17						
IgA ^3^, μg/mL	110.82 ^a^	75.64 ^b^	86.30 ^b^	111.60 ^a^	3.59	<0.001
IgG ^3^, mg/mL	7.07 ^a^	5.54 ^b^	5.56 ^b^	6.52 ^a^	0.15	<0.001
IgM ^3^, mg/mL	2.39 ^a^	1.95 ^b^	2.30 ^ab^	2.65 ^a^	0.07	0.004
IL-1β ^3^, pg/mL	95.35 ^b^	124.71 ^a^	87.92 ^b^	90.12 ^b^	3.15	<0.001
IL-6 ^3^, pg/mL	258.54 ^b^	293.29 ^a^	236.58 ^c^	274.42 ^a,b^	4.55	<0.001
TNF-α ^3^, pg/mL	49.81 ^b^	56.24 ^a^	48.89 ^b^	52.43 ^a,b^	1.06	0.056
Days 42						
IgA, μg/mL	110.00 ^b^	100.63 ^b^	106.80 ^b^	126.88 ^a^	2.73	0.002
IgG, mg/mL	6.79 ^a^	6.01 ^b^	6.59 ^a^	6.93 ^a^	0.11	0.015
IgM, mg/mL	2.60 ^a,b^	2.24 ^b^	2.57 ^ab^	2.99 ^a^	0.08	0.012
IL-1β, pg/mL	93.89 ^a^	94.14 ^a^	70.97 ^b^	93.38 ^a^	2.31	<0.001
IL-6, pg/mL	243.58 ^a,b^	264.04 ^a^	222.88 ^b^	244.54 ^a^	4.01	0.001
TNF-α, pg/mL	49.20 ^a,b^	50.64 ^a^	45.52 ^b^	50.26 ^a,b^	0.83	0.108

^a–c^ Different superscript letters within a row indicate significant difference between groups (*p* < 0.05). ^1^ Dietary treatments were as follows: NC, negative control group, basal diet; PC, positive control group, basal diet, and challenged with *E. coli* K88; AGP, antibiotic growth promoter group, basal diet supplemented with 75 mg/kg chlortetracycline, 50 mg/kg oxytetracycline calcium, and 40 mg/kg zinc bacitracin, and challenged with *E. coli* K88; IPM, IgY and PM group, basal diet supplemented with IgY at dose of 2.5 g/kg and 1 g/kg and PM at dose of 300 mg/kg and 150 mg/kg during days 1 to 17 and 18 to 42, respectively, and challenge with *E. coli* K88. ^2^ SEM, standard error of the mean, *n* = 8. ^3^ IgA, immunoglobulin A; Ig M, immunoglobulin M; Ig G, immunoglobulin G; IL-1β, interleukin 1β; IL-6, interleukin; TNF-α, tumor necrosis factor.

**Table 5:** Effect of dietary treatments on the intestinal morphology of *E. coli* K88-challenged weaned pigs.

Items	Dietary Treatments ^1^				SEM ^2^	*p*-Value
	NC	PC	AGP	IPM		
Duodenum						
VH ^3^, µm	572.95 ^b^	563.88 ^b^	642.81 ^a^	612.36 ^a,b^	9.91	0.010
CD ^3^, µm	280.27	320.02	306.79	291.34	7.12	0.216
V/C ^3^	2.07 ^a^	1.77 ^b^	2.12 ^a^	2.12 ^a^	0.05	0.008
Jejunum						
VH, µm	598.56 ^ab^	529.59 ^b^	563.28 ^b^	647.48 ^a^	13.81	0.011
CD, µm	273.01	288.97	264.57	263.11	7.11	0.575
V/C	2.21 ^b^	1.84 ^c^	2.13 ^b^	2.49 ^a^	0.05	<0.001
Ileum						
VH, µm	528.11 ^a,b^	457.48 ^b^	560.95 ^a^	559.91 ^a^	16.15	0.071
CD, µm	233.33	215.87	241.21	243.46	7.29	0.550
V/C	2.30	2.13	2.37	2.30	0.05	0.117

^a,b^ Different superscript letters within a row indicate significant difference between groups (*p* < 0.05). ^1^ Dietary treatments were as follows: NC, negative control group, basal diet; PC, positive control group, basal diet, and challenged with *E. coli* K88; AGP, antibiotic growth promoter group, basal diet supplemented with 75 mg/kg chlortetracycline, 50 mg/kg oxytetracycline calcium, and 40 mg/kg zinc bacitracin, and challenged with *E. coli* K88; IPM, IgY and PM group, basal diet supplemented with IgY at dose of 2.5 g/kg and 1 g/kg and PM at dose of 300 mg/kg and 150 mg/kg during days 1 to 17 and 18 to 42, respectively, and challenge with *E. coli* K88. ^2^ SEM, standard error of the mean, *n* = 8. ^3^ VH, villi height; CD, crypt depth, V/C, the ratio of villi height to crypt depth.

## Data Availability

The data presented in this study are not publicly available due to privacy restrictions.
